# Nurse‐led remote digital support for adults with chronic conditions: A systematic synthesis without meta‐analysis

**DOI:** 10.1111/jocn.17226

**Published:** 2024-06-18

**Authors:** Alicia Kilfoy, Charlene Chu, Archanaa Krisnagopal, Enoch Mcatee, Sunny Baek, Mallory Zworth, Kyobin Hwang, Hyun Park, Lindsay Jibb

**Affiliations:** ^1^ Lawrence S. Bloomberg Faculty of Nursing University of Toronto Toronto Ontario Canada; ^2^ Division of Hematology/Oncology Hospital for Sick Children Toronto Ontario Canada; ^3^ Child Health Evaluative Sciences Hospital for Sick Children Toronto Ontario Canada; ^4^ KITE Research Institute University Health Network Toronto Ontario Canada; ^5^ Present address: Casey House Toronto Ontario Canada; ^6^ Present address: Holland Bloorview Kids Rehabilitation Hospital Toronto Ontario Canada

**Keywords:** adult nursing, chronic illness, digital, nursing intervention, self‐management, support

## Abstract

**Aim:**

The systematic review aims to synthesize the literature examining the effectiveness of nurse‐led remote digital support on health outcomes in adults with chronic conditions.

**Background:**

Adults with chronic diseases have increased rates of mortality and morbidity and use health care resources at a higher intensity than those without chronic conditions—placing strain on the patient, their caregivers and health systems. Nurse‐led digital health disease self‐management interventions have potential to improve outcomes for patients with chronic conditions by facilitating care in environments other that the hospital setting.

**Design and Methods:**

We searched PubMed/MEDLINE, Embase, PsycINFO and Cochrane Central databases from inception to 7 December 2022. We included randomized controlled trials assessing the impact of nurse‐led remote digital support interventions compared to usual care on health‐related outcomes in adults with chronic illness. The Cochrane risk‐of‐bias tool was used to assess bias in studies. Outcomes were organized into four categories: self‐management, clinical outcomes, health care resource use and satisfaction with care. Results are presented narratively based on statistical significance.

**Results:**

Forty‐four papers pertaining to 40 unique studies were included. Interventions most targeted diabetes (*n* = 11) and cardiovascular disease (*n* = 8). Websites (*n* = 10) and mobile applications (*n* = 10) were the most used digital modalities. Nurses supported patients either in response to incoming patient health data (*n* = 14), virtual appointment (*n* = 8), virtual health education (*n* = 5) or through a combination of these approaches (*n* = 13). Positive impacts of nurse‐led digital chronic disease support were identified in each outcome category. Mobile applications were the most effective digital modality.

**Conclusion and Relevance to Clinical Practice:**

Results show that nurse‐led remote digital support interventions significantly improve self‐management capacity, clinical health outcomes, health care resource use and satisfaction with care. Such interventions have potential to support overall health for adults with chronic conditions in their home environments.

**What does this paper contribute to the wider global clinical community?**

- This review provides insight into the potential for nurse‐led digital interventions to support the health of adults with chronic conditions
- This review outlines key characteristics of identified interventions which can be used as a guide for future intervention development

## 1 INTRODUCTION

The prevalence of chronic health conditions such as hypertension, diabetes and cancer, is increasing globally due to an overall aging population and advancements in health care and treatment which have improved mortality rates (Ansah & Chiu, 2023). In Canada, the number of adults with a chronic condition increased by 11% over the last decade—to 10 million adults (Steffler et al., 2021). Chronic diseases and their treatments have a significant impact on the patient, their family, particularly those family members in caregiving roles, and health systems and other sectors (Walker et al., 2019). Patients with chronic diseases have increased rates of mortality and morbidity, reduced quality of life and spend more time in a hospital setting compared to the general public (Golics et al., 2013; Walker et al., 2019). The common requirement for acute medical and psychosocial support for those with chronic conditions also strains the health system in terms of staffing and other resources and ultimately can be economically burdensome (Jeon et al., 2009). Interventions that support chronic disease self‐management, prevent deterioration and hospitalizations have great potential to support the best possible patient and family quality of life while enhancing health system function.

Remote, digitally enabled support interventions may fit this role and can enhance health outcomes for patients with chronic conditions, including in their own home environments (Hanley et al., 2015). These interventions involve the digital linking of a device (mobile phone application, tele‐monitoring device) between the patient and their electronic clinical data to a clinician or a clinical site (WHO, 2018). Studies to date have shown that these interventions can increase patient disease self‐management (Hanley et al., 2015; Ure et al., 2011) and quality of life and decrease mortality rates (Nakamura et al., 2014). In addition, such interventions decrease the rates of hospital admissions for those with chronic conditions, improve patient care satisfaction and build health system capacity (McLean et al., 2012).

Familiarity and expertise in patient supportive care means nurses are well poised to bolster the impact of these interventions in adults with chronic conditions (Griffin, 2017). Qualitative studies show that nurse engagement in health care and self‐management interventions is a sought‐after clinical feature by patients and caregivers (Jibb et al., 2018, 2021). Combining nursing care with digital health has the potential to create healthier communities and may improve patient health outcomes (Troncoso & Breads, 2021). Despite this potential, patients can be hesitant to use digital interventions due to privacy concerns (Madanian et al., 2023) and misconceptions regarding the benefits of these tools (Antes et al., 2021). In addition, there has been slow progress in nurses championing and leading digital health interventions due to a lack of leadership in the area and investment (Booth et al., 2021).

### 1.1 The review and aim(s)

Evidence around nurse involvement in digital chronic disease self‐management is needed to direct future intervention development and promote patient uptake of effective nurse‐engaged tools. The aim of this systematic review was then to identify and synthesize the scientific literature examining the effectiveness of nurse‐led digital interventions on health outcomes in adults with chronic health conditions.

## 2 METHODS/METHODOLOGY

### 2.1 Study design and inclusion/exclusion criteria

An internal, unregistered protocol for this systematic review was developed. The Cochrane handbook and the Synthesis Without Meta‐Analysis (SWiM) in Systematic Reviews guideline guided the reporting (Campbell et al., 2020). Randomized control trials (RCTs) published in peer‐reviewed journals with an experimental group that received a nurse‐led digital remote chronic disease support intervention and with a control group that received usual medical care were included. Nurse‐led remote digital health interventions were defined as interventions where a nurse remotely monitored a patient's chronic health condition, defined as conditions lasting >3 months and digitally supported the patient in managing that condition while living in the community (The Dutch National Consensus Committee Chronic Diseases and Health Conditions in Childhood et al., 2008). To be included, these interventions had to be delivered via a digital solution such as texting, mobile apps, tele‐care, or websites. Nurses in the studies were defined as any qualified healthcare professional working as any of a nurse practitioner, clinical nurse specialist, advanced practice nurse, practical nurse, registered nurse, registered practical nurse, or licensed practical nurse. Furthermore, nurses must have been responsible for the overall co‐ordination, management and continuity of care, but this was not exclusive of medical staff being present or participating.

To be included in the review, studies must have explored the impact of the intervention on at least one observer‐ or patient‐reported health outcome. Observer‐reported outcomes were those that were rated and scored by someone other than the patient and included biochemical or physiological measurements (i.e. blood pressure, heart rate, blood glucose). Patient‐reported outcomes were defined as those relating to patient health or care satisfaction.

### 2.2 Search methods, search outcome and data abstraction

The search strategy was developed by a health sciences librarian and nurse team members with research experience in digital health and focused on the concepts of nurses as health care professionals and telemedicine. The search was conducted on 7 December 2022, in Cochrane Central, Embase, MEDLINE, PsycINFO. The search strategy is available in Appendix S1. All retrieved results were uploaded to Covidence for screening and duplicates were removed. Independent reviewers screened titles and abstracts and full‐text records for eligible studies. Disagreements were resolved through discussion with a third author.

A standardized data extraction form was developed and piloted with three articles. No revisions to the form were necessary. Data extraction was completed independently by team members and reviewed for accuracy.

### 2.3 Quality appraisal

Seven reviewers assessed the risk of bias using criteria from the Cochrane risk‐of‐bias tool for randomized trials (ROB2) (Sterne et al., 2019). A random sample of 10% of bias ratings for outcomes within each study were conducted induplicate by secondary reviewers. If discrepancies in ratings were greater than 10% between the reviewers, all outcomes for that study were assessed in duplicate and disagreements resolved through group arbitration. Images for ROB2 were adapted from images made with ROBVIS (McGuinness & Higgins, 2020).

### 2.4 Outcomes and data synthesis

Given the broad scope of the review and the considerably high heterogeneity of studies and outcomes, data was summarized using narrative synthesis, tabulation and descriptive analysis. To synthesize the cumulative impact of nurse‐led digital support interventions, outcomes were included in the narrative synthesis only if they were reported in two or more studies. Several other outcomes were identified and presented in tabular form. Study outcomes were classified into four major domains: patient self‐management, clinical outcomes, resource use and satisfaction with care. Significant impact of the intervention on outcomes was determined based on a reported statistical difference of *p* < 0.05 of either between group differences over time, between group differences, or a within group pre‐ to post‐test difference. Based on clinical experience, trends were reported related to intervention effectiveness in each outcome domain.

## 3 RESULTS/FINDINGS

**FIGURE 1:**
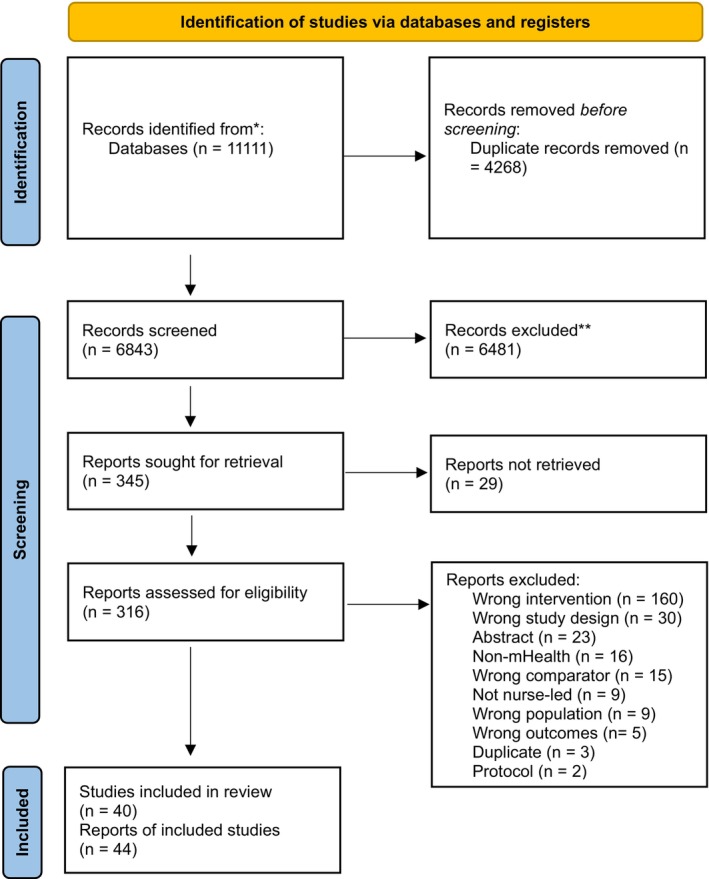
PRISMA flowchart of studies included in the review. This flowchart shows the number of records identified from the search (6843 non‐duplicative records), the number of records excluded based on title and abstract (6481) and the number of studies excluded based on the full article review (316), and the reason for exclusions. Forty‐four research articles (about 40 studies) were included in this analysis. [Colour figure can be viewed at wileyonlinelibrary.com]

The literature search identified 11,111 articles. After excluding duplicates, 6843 titles and abstracts were screened for inclusion. Full texts of 345 articles were screened for eligibility and excluded 301. Forty‐four articles, reporting on 40 unique studies, were included in the final analysis (Figure 1).

### 3.1 Characteristics and participant traits in included studies

Table 1 presents the characteristics of the 40 included studies. Studies were published between 2003 and 2022 in 16 different countries, most often in the United States (11/40; 38%) and China (6/40; 15%). Thirty‐seven randomized controlled trials (RCTs) were two‐arm studies (92.5%), two were three‐arm studies (5%) and one was a four‐arm study (2.5%). Of those with multiple intervention groups, studies commonly included a non‐digital health arm involving care modalities such as telephone calls, in‐person interventions or varied the intensity of digital intervention across arms. Study sample size ranged from 45 to 1665 with an average size of 205 (SD = 266) participants. Study participants age ranged from 18 to 82 years. Diabetes was the most common chronic health condition targeted (11/40; 28%); with seven (18%) studies focused on type two diabetes, two (5%) studies focused on both type one and type two diabetes, and two (5%) studies targeting gestational diabetes. Eight studies (20%) enrolled individuals with cardiovascular disease including hypertension (two studies) and heart failure (two studies). Three studies aimed to address each of cancer (8%) and mental health (8%), and two studies targeted each of obesity (5%), asthma (5%) and musculoskeletal disorders (5%). Chronic kidney disease (CKD), neurological disorders and chronic obstructive pulmonary (COPD) disease were targeted each by one study (3%). Last, six studies (15%) enrolled participants with a range of chronic disorders including cardiovascular disorders, diabetes and respiratory issues.

**TABLE 1:** Study, participant and intervention characteristics.

Study		Participants			Nurse‐led digital intervention			Outcomes	
Study and location	Study design/duration	Health condition	Intervention group	Control group	Type and length	Structure and content	Details of nurse contact	Outcome type	Assessment timepoints
Ahmed et al., 2016 (Canada)	RCT, 9 months	Asthma	*n* = 49, age reported in ranges. 18–39 years old: *n* = 32 (43%), 40–40 years old: *n* = 10 (21%), 50–59 years old: *n* = 12 (26%), 60–69 years old: *n* = 5 (11%), 32/47 (68%) of participants were female	*n* = 51, age reported in ranges. 18–39 years old: *n* = 14 (27%), 40–49 years old: *n* = 13 (25%), 50–59 years old: *n* = 15 (39%), 60–69 years old: *n* = 9 (18%), 33/51 (65%) of participants were female	Combination of remote digital support, 6 months	Participants logged into online portal weekly with tailored health information, asthma education, and where they could monitor and receive feedback on self‐management from a nurse if needed	Nurses responded to participants as needed	Asthma control, asthma quality of life, chronic disease self‐efficacy, asthma control, beliefs about medicines patient health, Asthma‐related ED visits/hospitalizations	3,6,9 months
Arad et al., 2021 (Iran)	RCT, 6 months	Chronic kidney disease	*n* = 33, mean age in years (SD) = 27 (11.5), 15/33 (45.5%) of participants were female	*n* = 33, mean age in years (SD) = 30 (9.5), 14/33 (42.4%) of participants were female	Scheduled virtual appointments, 3 months	Participants received a patient education program and nurse led follow‐up services through telephone communication and the Short Message Service (SMS)	Nurses sent text messages daily and contacted participants twice a week on the phone	Haemodialysis (HD) attendance, medication adherence, fluid restrictions, diet recommendations, total treatment adherence	3,4,6 months
Bell et al., 2012 (United States)	RCT, 1 year	Type 1 and 2 diabetes	*n* = 31, mean age in years (SD) = 55 (10), 16/31 (52%) participants were female	*n* = 33, mean age in years (SD) = 60 (11), 13/33 participants were female	Virtually enabled nurse health education, 6 months	Nurse Practitioners (NPs) created 30–60 second videos covering self‐care topics which were sent to participants through SMS in random order and could be viewed multiple times throughout the 24‐h period before the next video sent	NPs sent asynchronous videos daily	Glycemic control, if clients reported self‐monitoring blood glucose (SMBG) measurements, the proportion of SMBG measurements above 180 mg/dL and below 70 mg/dL, mean SBMG values at each quarterly visit	3, 6, 9, 12 months
Chen et al., 2022 (Taiwan)	RCT, 6 months (length varied based on pregnancy)	Obesity/gestational weight gain	*n* = 46, mean age equal or under 35 years in years (%) = 31 (67.4), mean age over 35 years 15 (32.6), all participants were female	*n* = 46, mean age equal or under 35 years in years (%) = 35 (76.1), mean age over 35 years 11 (23.9), no sex ratio reported	Combination of remote digital support, 6 months	Participants had access to an app and a wrist‐worn monitoring ban. Through the app they could set goals, self‐monitor, self‐evaluate, self‐reward and receive personalized educational text messages	Nurses designed weekly, educational text messages	Gestational weight gain	3, 6 months (second and third trimester)
Cicolini et al., 2014 (Italy)	RCT, 6 months	Hypertension	*n* = 100, mean age in years (SD) = 59.8 (15.0), 50/100 (50.0%) participants were female	*n* = 98, mean age in years (SD) = 58.3 (13.9), 46/98 (47%) of participants were female	Virtually enabled nurse health education, 6 months	Nurse case managers sent emails to participants reinforcing the important of a healthy lifestyle to participants weekly	Nurses contacted participants weekly	Blood pressure, LDL, HDL, TG, glycemia, BMI, alcohol consumption, smoking, fruit and vegetable intake, salt intake, physical activity, adherence to antihypertensive therapy	3, 6 months
Egede et al., 2020; Egede et al., 2021 (United States)	RCT, 6 months	Type 2 diabetes	*n* = 54, mean age in years (SD): 55.1 (11.4), 44/54 (81.4%) of participants were female	*n* = 59, mean age in years (SD): 53.4 (10.5), 48/59 (81%) of participants were female	Data sent to RN for review, 6 months	Participants sent nurses blood glucose and blood pressure measurements daily through a FORA system and the internet. In response, nurses would call participants to change or titrate medications	Nurses contacted participants weekly or bi‐weekly if needed	HbA1c, blood pressure, quality of life	3, 6 months
Greving et al., 2015; Vernooij et al., 2012 (The Netherlands)	RCT, 12 months	Vascular disease	*n* = 164, mean in years (SD) = 60.7 (7.8), 36/164 (22%) participants were female	*n* = 166, mean age in years (SD) = 59.2 (8.9), 48/166 (29%) of participants were female	Scheduled and as needed virtual appointments with a nurse, 12 months	Participants had access to a personalized website which described their own risk factors, drug use, treatment goals and allowed for correspondence with NPs	At minimum nurses contacted participant biweekly and as needed	Quality‐adjusted life‐years, 10‐ year risk for coronary heart disease, body mass index, total cholesterol, HDL, triglycerides, LDL, glucose, HbA1C, systolic blood pressure, diastolic blood pressure, EGFR, albuminuria	3, 6, 9, 12 months
Jiang et al., 2021 (Singapore)	RCT, 6 months	Heart failure	*n* = 57, mean age in years (SD) = 66.82 (11.81), 17/57 (29.8%) of participants were female	*n* = 56, mean age in years (SD) = 68.82 (13.14), 19/56 (33.9%) of participants were female	Combination of remote digital support, 6 weeks	In addition to receiving a home‐based multi‐component self‐management intervention, the participants had access to an app with educational information, individualized scheduled reminders, weight and blood pressure logs and nurse chat	Nurses would review data and respond as needed	HF self‐care in terms of maintenance, management, and confidence; cardiac self‐efficacy, anxiety, depression, health‐related Quality of Life, social support. Satisfaction, HF severity, number of unplanned hospital admissions, unplanned A&E visits, unplanned medical consultations	6 weeks, 3, 6 months
Jiang et al., 2022 (Singapore)	RCT, 6 months	Type 2 diabetes	*n* = 58, mean age in years (SD): 49.6 (12.43). 21/58 (36%) of participants were female	*n* = 56, mean age in years (SD): 56 (9.59). 19/56 (34%) of participants were female	Combination of remote digital support, 6 months	Participants had access to an app with self‐help educational resources and recording of their blood glucose, diet and exercise. The nurse could view their clinical data and counsel patients as needed	Nurses would review data daily and responded	Self‐efficacy, diabetes self‐care, quality of life, diet (general and specific), exercise, blood glucose monitoring, foot care, HbA1c, number of unplanned hospital admissions, unplanned medical consultations, and unplanned emergency department visits	3, 6 months
Kenealy et al., 2015 (New Zealand)	RCT, 6 months	CHF, COPD and Type 2 diabetes	*n* = 98 (Site A *n* = 49, Site B *n* = 24, Site C *n* = 25). Mean age reported by site. Site A: 72 years old, Site B: 67 years old, Site C: 57 years old. Sex ratio reported by site. Site A: 19/49 (39%) participants were female, Site B: 9/24 (38%) participants were female, Site C: 15/25 (60%) participants were female	*n* = 73 (Site A *n* = 49, Site B *n* = 24), average age reported by site. Site A: 72 years, Site B: 67.5 years. Note no UC recruited from Site C. Sex ratios reported by site. Site A: 14/49 (29%) participants were female, Site B: 9/24 (38%) participants were female	Data sent to nurse for review, 6 months	Participants entered various health data manually into a health hub which facilitated transfer of data, through landline, to a nurse who assessed data daily and would record a response to abnormal data in the system. Nurses would also call participants if needed	Nurses reviewed data daily and responded	Physical and Mental component score, anxiety, depression, self‐efficacy, respiratory self‐reported symptoms, activity and impacts, diabetes self‐care activities, count and length of hospital admissions, ER visits, outpatient visits and deaths	3, 6 months
Kes et al., 2022 (Turkey)	RCT, 12 weeks	Hypertension	*n* = 39, mean age in years (SD): 54.9 (6.6). 20/39 (51.3%) of participants were female	*n* = 38, mean age in years (SD): 52.2 (6.2). 21/38 (55.3%) of participants were female	Virtually enabled nurse health education, 12 weeks	Participants received in‐person training regarding hypertension, follow up‐telephone calls and one‐way, personalized text messages with education and medication reminders	Nurse sent personalized text messages 3 times for 5 weeks	Blood pressure, medication adherence	12 weeks
Kim et al., 2007, Kim et al., 2007 (South Korea)	RCT, 6 months	Type 2 diabetes	*n* = 25, mean age in years (SD): 46.8 (8.8). 14/25 (56%) of participants were female	*n* = 26, mean age in years (SD): 47.5 (9.1), 15/26 (57%) of participants were female	Data sent to nurse for review, 6 months	Patients sent glucose levels and drug information to a nurse through a website. Nurses would send recommendations back to each patient weekly by a SMS	Nurses reviewed data weekly and responded	Blood glucose levels in a normal range	3, 6 months
Lyu et al., 2021 (China)	RCT, 3 months	Type 2 diabetes	*n* = 54, mean age in years (SD): 60.0 (10.02). 26/54 (48%) of participants were female	*n* = 52, mean age in years (SD): 61.69 (10.54). 29/52 (56%) of participants were female	Combination of remote digital support, 3 months	Participants has access to a web‐based transitional care program including disease self‐management, health education, group interaction, remote counselling, and data collection	Nurses provided 24‐hour online consultation service and sent educational content based on treatment compliance	Glycemic control, quality of life, self‐efficacy, treatment adherence, physical component score, mental component score	3 months
Liang et al., 2021 (Taiwan)	RCT, 6 months	Multiple chronic illnesses	*n* = 100, mean age in years (SD) = 79.83 (6.54). 59/100 (59%) of participants were female	*n* = 100, mean age in years (SD) = 81.51 (7.91). 57/100 (57%) of the participants were female	Data sent to nurse for review, 6 months	Participants transferred health data twice daily to a nurse through a Bluetooth‐enabled smartphone. Nurses would review data immediately and call participants to counsel them as needed	Nurses reviewed data daily and responded as needed	Mortality, readmission number of ED visits, medication adherence, ADLs, perceived health status, quality of life	3, 6 months
Metilda et al., 2021 (India)	RCT, 5 months	Chronic neurosurgical disorders	*n* = 50, mean age in years (SD), 40.44 (14.7) 20/50 (40%) of participants were female	*n* = 50, mean age in years (SD), 40.4 (15.8), 19/50 (38%) of participants were female	Combination of remote digital support, 2 months	Participants were provided with a mobile phone app which held an individualized discharge summary with educational videos. Patients were able to connect with nurses through the app if concerns arose	Nurses contacted participants as needed	Medication compliance, lifestyle practices, revisits	3 months
Mir et al., 2022 (France)	RCT, 6 months	Cancer	*n* = 272, age reported in ranges. <45 years old: *n* = 42 (15.4%), 45–54 years old: *n* = 45 (16.5%), 55–64 years old: *n* = 70 (25.7%), 65–74 years old: *n* = 76 (27.9%), ≥75 years old: *n* = 39 (14.3%). 156/272 (57.4%) of participants were female	*n* = 287, age reported in ranges. <45 years old: *n* = 37 (12.9%), 45–54 years old: *n* = 49 (17.1%), 55–64 years old: *n* = 83 (28.9%), 65–74 years old: *n* = 79 (27.5%), ≥75 years old: *n* = 39 (13.6%). 174/287 (60.6%) of participants were female	Combination of remote digital support, 6 months	The web page allowed nurses to monitor records of patients, create interaction reports, share interactions with other healthcare professionals and interact with the patients directly	Nurses could monitor clinical data and held scheduled appointments: weekly for the first month, biweekly for 3 months and every 3 weeks for the final 2 months. Nurses could also be contacted as needed during weekday hours	Optimization of treatment dose, grade ≥3 toxicity, patient adherence to treatment, patient experience, tumour response, progression‐free survival, overall survival	1, 2, 3, 4, 5, 6 months
Mumcu et al., 2022 (Turkey)	RCT, 6 months	Type 2 diabetes	*n* = 31, 65% of the intervention group was between 50 and 65 years old, all participants were female	*n* = 30, 70% of the control group was between 50 and 65 years old, all participants were female	Virtually enabled nurse health education, 6 months	The website contained six educational modules and videos which were published sequentially during the 6‐ month period	Nurse Practitioners created content for the educational website	Diabetes self‐care, diabetes family support and conflict, metabolic control	3, 6 months
Pakrad et al., 2021 (Iran)	RCT, 4 months	Cardiovascular disease	*n* = 44, mean age in years (SD) = 62.6 (8.1). 8/44 (18.2%) of participants were female	*n* = 44, mean age in years (SD) = 62.9 (9.8). 6/44 (13.6%) of participants were female	Combination of remote digital support, 3 months	The app included educational content and provided a means for patients to communicate with the nurse	Nurses reviewed data biweekly and responded	Quality of life, functional capacity, psychological well‐being (depression, anxiety, and stress), re‐hospitalization	1, 3, 4 months
Parker et al., 2022 (Australia)	RCT, 12 months	Obesity	*n* = 120, mean age in years (SD) = 58.9 (8.8). 60/120 (50%) of participants were female	*n* = 95, mean age in years (SD) = 56.2 (9.6). 32/95 (34%) of participants were female	Combination of remote digital support, 12 months	The app allowed participants to set and revise goals, view progress, educational resources, and text‐message reminders regarding follow‐up. In addition, nurses provided coaching over the telephone and in person health checks	Nurses conducted health‐checks and follow‐ups. Nurses developed the written and video resources for the app	Health literacy, eHealth literacy, weight, waist circumference, blood pressure, diet, physical activity, blood lipids, quality of life	6, 12 months
Ritchie et al., 2016 (United States)	RCT, 30 days	CHF and COPD	*n* = 233, mean age in years (SD) = 63.0 (12.1). 124/233 (53.2%) of participants were female	*n* = 245, mean age in years (SD): 63.8 (12.8), 103/245 (42.0%) of participants were female	Data sent to nurse for review, 30 days	An interactive voice response system called participants daily and ask a series of health‐related questions. This data would be transferred a nurse's computer who would call participants back if needed	Nurses reviewed data daily and responded	30‐day rehospitalization, death	30 days
Sawyer et al., 2019 (Australia)	RCT, 12 months	Postnatal depression	*n* = 54, mean age in years (SD) = 31.1 (5), all participants were female	*n* = 57, mean age in years (SD) = 32.2 (4), all participants were female	Combination of remote digital support, 4 months	Participants had access to a mobile phone application with various resources and education components and a chat function where nurses would respond to questions posted by mothers. Nurses could also respond privately to questions	Nurses responded to participants as needed	Maternal depressive symptoms, the quality of maternal caregiving including the level of parenting self‐competence, the quality of the mother‐infant relationship	When infants were 8, 12 months
Shea et al., 2006, Shea et al., 2009 (United States)	RCT, 5 years	Type 1 and 2 diabetes	*n* = 844, mean age in years (SD) = 70.8 (6.5), 536/844 (63.5%) of participants were female	*n* = 821, mean age in years (SD) = 70.9 (6.8), 510/821 (62.1%) of participants were female	Data sent to nurse for review, 5 years	Participants sent heath data to a nurse through a telemedicine unit via a landline. The unit also included a webcam where nurses could videophone participants as in response to data	Nurses would review data daily and responded as needed	HbA1C, LDL, cholesterol, blood pressure	1, 2, 3, 4, 5 years
Simon et al., 2011 (United States)	RCT, 6 months	Depression	*n* = 106, mean age in years (SD) = 46 (13), 73/106 (69%) participants were female	*n* = 102, mean age in years (SD) = 45 (14), 77/102 participants were female	Scheduled virtual appointments, 10 weeks	Nurses sent participants an online questionnaire to fill out at scheduled times and would reply to participant based on results	Nurses sent questionnaire to participants 2, 6 and 10 weeks after initial on‐boarding	Depression, satisfaction, use of health services, filled anti‐depressant prescriptions, outpatient visits	5, 6 months
Simsek‐Cetinkaya et al., 2022 (Turkey)	RCT, 14 weeks	Gestational diabetes	*n* = 23, age reported in ranges. 18–34 years old: *n* = 15 (65%), 35–45 years old: *n* = 8 (35%), all participants were female	*n* = 22, age reported in ranges. 18–34 years old: *n* = 17 (77%), 35–45 years old: *n* = 5 (23%), all participants were female	Combination of remote digital support,14 weeks	Participants received a mobile app which consistent of digital education, health tracking. Through the app they also received individual and group counselling	Nurses provided education along with individual and group counselling weekly	Adherence to diet, blood glucose, insulin therapy, satisfaction, physical activity, gestational diabetes mellitus knowledge	14 weeks
Sorknaes et al., 2013 (Denmark)	RCT, 26 months	Acute exacerbation of chronic obstructive pulmonary disease (AECOPD)	*n* = 132, age in years (SD) = 71 (10), 79/132 (60%) of participants were female	*n* = 134, mean age (SD) = 72 (9), 83/134 (62%) participants were female	Scheduled virtual appointments with nurse, 5–9 days	Nurses called participant daily using a videophone platform to discuss discharge education	Nurses called daily	Total readmission, AECOPD readmission, total hospital days, AECOPD hospital days	4, 8, 12, and 26 weeks
Southard et al., 2003 (United States)	RCT, 6 months	Cardiovascular disease	*n* = 53, mean age in years (SD) = 61.8 (10.6), 17/53 (32%) of participants were female	*n* = 51, mean age in years (SD) = 62.8 (10.6), 9/51 (18%) of participants were female	Combination of remote digital support, 6 months	Participants had access to a website where they could access educational videos and modules, track their progress and communicate with a nurse	Nurse case managers communicated with participants a minimum of once a week or more frequently if needed	Risk of cardiovascular events, weight loss, blood pressure, depression scores, minutes of exercise and dietary habits	6 months
Su & Yu, 2021 (China)	RCT, 24 weeks	Coronary heart disease	*n* = 73, mean age in years (SD) = 55.53 (7.30), 11/73 (15%) of participants were female	*n* = 73, mean age in years (SD) = 56.03 (7.02), 13/73 (17.8%) of participants were female	Combination of remote digital support, 12 weeks	Participants had access to a website where they could enter goals focused on self‐management, view educational components, and post on a health dialogue forum. Nurses could review data and respond to participants using WeChat	Nurses reviewed data weekly and responded	Physical activity, health‐promoting lifestyle habits, smoking status, pedometer measurements, cardiac self‐efficacy, health‐related quality of life, psychosocial wellbeing, and cardiac physiological risk factors	18 and 24 weeks
Su et al., 2021 (Taiwan)	RCT, 6 months	Gestational diabetes	*n* = 56, mean age in years (SD) = 35.7 (4.3), All participants were female	*n* = 56, mean age in years (SD) = 35.8 (4.3), All participants were female	Combination of remote digital support, 6 months	Participants received a web‐based health management program where they were encouraged to log their health information. They also tailored health education consultations via LINE mobile app	Nurses facilitated the web‐based health management program and review data a minimum of once a week	Diastolic and systolic pressure, triglycerides, HDL cholesterol, total cholesterol, incidence of MS, weight, waist circumference, metabolic syndrome, BMI	3, 6 months
Tang et al., 2013 (United States)	RCT, 12 months	Type 2 diabetes	*n* = 202, mean age in years (SD) = 54.0 (10.7), 83/202 (41.1%) participants were female	*n* = 213, mean age in years (SD) = 53.5 (10.2), 83/213 (39%) participants were female	Data sent to nurse for review, 12 months	Participants had access to an online platform with personalized actions plans, their electronic health record, a portal to upload glucose readings, a chat function with a nurse	Nurses contacted participants as needed based on glucose readings and tailored educational goals	Glycemic control, weight, blood pressure, if clients reported self‐monitoring blood glucose ( measurements, the proportion of SMBG measurements above 180 mg/dL and below 70 mg/dL	6, 12 months
Tonning et al., 2021 (Denmark)	RCT, 6 months	Unipolar depressive disorder	*n* = 59, mean age in years (SD) = 44.5 (14.0), 28/59 (47.5%) of the participants were female	*n* = 61, mean age in years (SD) = 43.4 (14.3), 35/61 (57.4%) of the participants were female	Combination of remote digital support, 6 months	Participants answered self‐rating questions daily on their smartphone which was transferred data to a nurse. Nurses reviewed data three times a week and would respond accordingly. Smartphone based CBT was also provided via SMS and small cartoons, included in the mobile phone system	Nurses reviewed data 3 times a week and responded	Rates/duration of readmission, severity of depressive symptoms, psychosocial functioning, number of depressive episodes, perceived stress, quality of life, adherence to medication, wellbeing, rumination, worrying, satisfaction with care	3, 6 months
Valdivieso et al., 2018 (Spain)	RCT, 12 months	Multiple chronic conditions in older patients	*n* = 95, mean age in years (SD) =68.83 (11.2), 28.42/95 (30%) participants were female	*n* = 198, mean age in years = 75.97, 45.96/198 (23%) of participants were female	Data sent to nurse for review, 12 months	Patients were provided with a Bluetooth enables tablet which facilitated transfer of health data to a nurse's computer for review. Nurses review the data and respond appropriately	Nurses contacted stable participants weekly for the first 10 weeks, and every two weeks afterwards. For unstable patients, nurses would contact participant as needed based on data	Health‐related quality of life, mortality, emergency and planned visits to the hospital, hospital admissions, health status, cognitive impairment status and patient performance	12 months
Wakefield et al., 2009 (United States)	RCT, 180 days	Chronic heart failure	*n* = 52 this study reported age and sex by entire population	*n* = 49	Scheduled virtual appointments with nurse, 90 days	Nurses contacted and assessed participants using a videophone platform. Based on assessment results, nurses reinforced the plan of care or made referrals to a physician	Nurses contacted participants three times the first week and then weekly for 11 weeks (14 contacts over 90 days)	Self‐efficacy, satisfaction with care, medication knowledge, compliance	90, 180 days
Wakefield et al., 2011 (United States)	RCT, 12 months	Type 2 diabetes and Hypertension	Low‐intensity intervention: *n* = 102, mean age in years (SD): 68.4 (9.5), 1/102 (1%) of participants were female. High‐intensity intervention: *n* = 93, mean age in years (SD): 67.8 (10), 1/93 (1%) of participants were female	*n* = 107, mean age in years (SD): 67.9 (9.9), 4/107 (3.7%) of participants were female	Data sent to nurse for review, 12 months	Participants sent health data to nurse through a telehealth device using a landline. Nurses responded to data as needed	Nurses reviewed data daily and responded	HbA1C, SBP	6, 12 months
Wakefield et al., 2014 (United States)	RCT, 3 months	Type 2 diabetes	*n* = 53, mean age in years (SD) = 57.7 (10.8). 28/53 (52.8%) of participants were females	*n* = 55, mean age in years (SD) = 62.5 (10.9). 32/55 (58%) of the participants were female	Data sent to nurse for review, 3 months	Participants used a Numera Net Connectivity Hub to transfer health data using a landline to a nurse daily or as frequently as they wanted. A nurse would review the data at least twice a week and provide tele‐coaching as needed	Nurses reviewed data twice a week and responded	HbA1C, SBP, DBP	3, 6 months
Wang et al., 2018 (China)	RCT, 6 months	Joint recovery after hip replacement	*n* = 194 mean age in years (SD) = 54.495 (13.553), 107/194 (55%) participants were female	*n* = 195, mean age in years (SD) = 56.841 (13.92). 100/195 (51%) participants were female	As needed virtual appointments with a RN, 6 months	Patients were provided with a mobile phone app where they could interact with a nurse upload photos/videos for review and book in‐person appointment	Nurses would chat with participants as needed	Functional joint recovery, quality of life, ADLs	3, 6 months
Wheelock et al., 2015 (United States)	RCT, 18 months	Breast cancer	*n* = 59, mean age in years (SD) = 54.87 (8.66), all participants were female	*n* = 41, mean age in years (SD) = 53.32 (10.79) all participants were female	Combination of remote digital support, 18 months	Participants received email invitations to complete an online questionnaire to document symptoms. An NP would review and call the patient to discuss. Patients could also request forms as needed	NPs contacted the participants every 3 months or more frequently if needed	Utilization of health care resources, number of physician visits	3,6, 9, 12 and 18 months
Willems et al., 2008 (Netherlands)	RCT, 12 months	Asthma	*n* = 55, mean age in years (SD) = 27.15 (19.3), 23/55 (41.8%) of participants were female	*n* = 54, mean age in years (SD) = 28.38 (21.0), 30/54 (55.6%) participants were female	Data sent to nurse for review, 12 months	Participants measured their daily peak flow twice a day using an electronic monitor. They were asked to send this data using a landline phone at least once a month to a nurse for review who responded appropriately	Nurses reviewed data daily and responded	Asthma specific quality of life, clinical asthma symptoms and medical consumption	4, 8 and 12 months
Wong et al., 2022 (China)	RCT, 6 months	Pain, Hypertension, Diabetes mellitus	*n* = 71, mean age in years (SD) = 77.63 (7.84), 62/71 (87.3%) of participants were female	*n* = 76, mean age in years (SD) = 77.38 (8.21), 63/76 (82.9%) of participants were female	Combination of remote digital support, 3 months	Participants used app to monitor vitals, schedule appointments, medication notifications, and receive education. Nurses monitored vitals and contacted participants when abnormalities were found within 24 hours	Nurses would review data daily and respond as needed	Quality of life, self‐efficacy, depression	3 and 6 months
Yan et al., 2022 (China)	RCT, 3 months	Osteoarthritic diseases	*n* = 50, mean age in years (SD) reported collectively with control group: 65.9 (6.81). 69/100 (69%) participants in total were female	*n* = 50, age and sex stratified by groups not provided	Virtually enabled nurse health education, 3 months	Participants received follow‐up, education and guidance through WeChat focused on pain, diet, psychology, medication, and functional training	Nurses sent health education via Internet and WeChat (frequency not discussed)	Compliance with follow‐up, total satisfaction, ADLs, pain, anxiety, depression, sleep quality	1, 3 months
Zhang et al., 2018 (China)	RCT, 25 weeks	Ovarian cancer	*n* = 36, age reported in ranges: 8–35 years old *n* = 2 (6.1%), 36–45 years old *n* = 6 (18.2%), 46–55 years old *n* = 14 (42.4%), 56–65 *n* = 11 (33.3%), all participants were female	*n* = 36, 18–35 years old *n* = 2 (5.9%), 36–45 *n* = 6 (17.6%), 46–55 *n* = 16 (47.1%), 56–65 *n* = 10 (29.4%), all participants were female	Scheduled virtual appointments with a nurse,12 weeks	Nurses performed scheduled 1 h CBT training sessions with participants via the internet	Nurse held sessions weekly	Fatigue symptoms, depression, and quality of sleep	3 and 6 months

### 3.2 Digital modality types involving nursing

A variety of different digital modalities were used to deliver the interventions. Websites and mobile applications were most used; by 10 studies each (25%). Nine (23%) studies utilized novel monitoring systems, four (10%) used text‐messaging, two (5%) used emails and two (5%) used video‐based platforms to connect patients in their homes to nurses. A portion of studies (3/40; 8%) used a combination of these modalities in their intervention. The type of intervention used by researchers evolved over time with monitoring systems, videophone platforms and basic websites commonly employed in the early 2000s; multi‐component websites, third‐party developed mobile applications and online messengers were employed commonly in the early 2010s; and investigator developed mobile applications and multi‐component websites being evaluated most recently.

### 3.3 Details of nurse‐led digital support

In 14/40 (35%) studies nurses contacted participants in response to incoming patient health data (i.e. blood glucose, blood pressure, weight, depressive symptoms). In studies where nurse contact was in response to such data, participant data was digitally transferred to a nurse's computer or telephone via a specialized ‘health‐hub’ device mediated by internet, Bluetooth, or landline connection (7/14; 50%), a smart‐phone (2/14; 14%), or information was recorded on a website (4/14; 29%). In the final study (1/14), an interactive voice service called participants to ask questions and participant data was transferred using a landline to a nurse's computer for review. In response to abnormal data, study nurses either called (7/14; 50%), text‐messaged (2/14, 14%), chatted through a mobile application (2/14; 14%), online messaged (1/14; 14%), video‐conferenced (1/14, 7%) or use a combination of these methods (1/14) with participants.

In eight of 40 (20%) studies, the nurse‐led intervention consisted of a virtual appointment with a nurse either at a scheduled time (3/8; 38%), as needed (3/8; 38%), or both (2/8; 25%). These appointments were facilitated through a mobile phone app (3/8; 38%); website (3/8; 38%), or videophone platform (2/8; 25%). Nurses provided virtual health education in five of 40 (13%) studies through a variety of digital modalities including mobile applications (2/5; 40%), emails (1/5; 20%), texting (1/5; 20%) and websites (1/5; 20%).

In the final 13/40 (33%) studies, nurses provided clinical support through a combination of these means. This included interventions where nurses created tailored health education for participants while simultaneously providing in‐the‐moment support through an online messenger. This approach to clinical support became increasingly more popular in recent years; with 10/13 (77%) studies published from 2018 onwards.

The frequency of nurse interaction with participants ranged from daily to no contact—in which case nurses were solely responsible for creating self‐management training content for the intervention. Most frequently, nurses interacted with participants (11/40; 28%) on an ‘as needed’ basis. This included online messaging the participant when the participant asked a question or answering participant questions on an online discussion board. Other frequently reported nurse contact schedules were daily (8/40; 20%) or weekly (8/40; 20%) and a portion of studies involved nurses connecting with participants twice a week (4/40; 10%), biweekly (1/40; 2.5%), monthly (1/40; 2.5%) and less than monthly (1/40; 2.5%). In addition, certain interventions also offered nurse support on a scheduled basis with additional support provided ad hoc, including two of 40 (5%) studies which offered support on a weekly basis and as requested and one of 40 (2.5%) studies which offered support biweekly and as requested. In three of 40 (7.5%) studies nurses were not in direct contact with patients and only created content for participants to view through digital modalities.

### 3.4 Outcomes of interest

**FIGURE 2:**
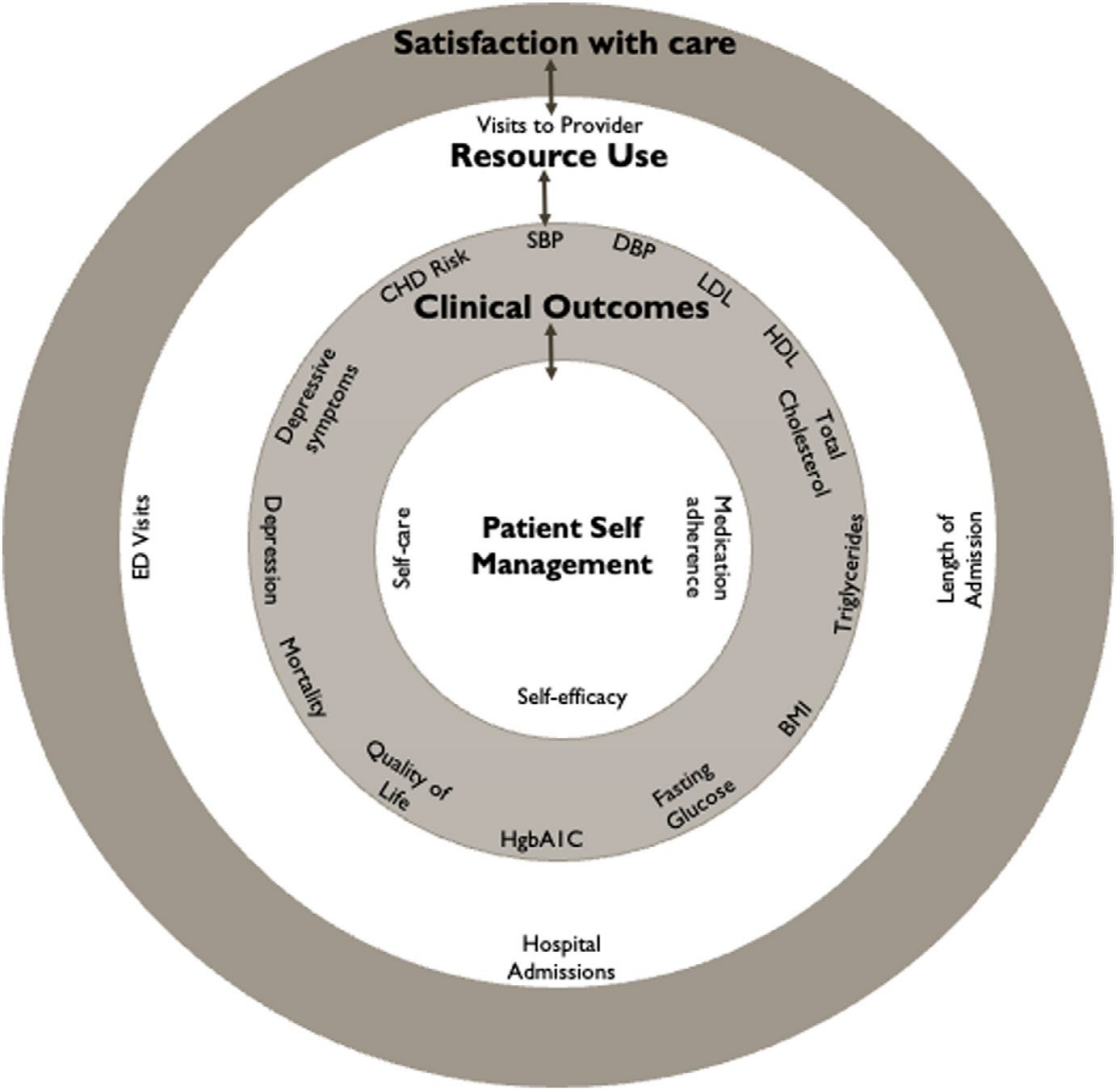
Framework for outcome synthesis. Identified outcomes were classified into four major headings: patient self‐management, (clinical outcomes, resource use and satisfaction with care. Included outcomes can be found in each category in the figure. Arrows indicate the relationships between categories. [Colour figure can be viewed at wileyonlinelibrary.com]

A proposed model detailing the relationships between study outcome domains is presented in Figure 2. A detailed list of these domains and their respected outcomes with results is shown in Table 2.

**TABLE 2:** Intervention effectiveness determined by a statistical significance of *p* < .05.

Core area	Outcome domain	Significantly positive patient impact	No significant patient impact	Significantly negative patient impact
Patient self‐management	Self‐efficacy	4/9 (44%)	5/9 (55%)	0/9 (0%)
	Self‐care	4/9 (44%)	5/9 (55%)	0/9 (0%)
	Treatment adherence	5/10 (50%)	5/10 (50%)	0/10 (0%)
Clinical outcomes	Depression	7/9 (78%)	2/9 (22%)	0/9 (0%)
	Depressive symptoms	0/2 (0%)	2/2 (100%)	0/2 (0%)
	Anxiety	2/2 (100%)	0/2 (0%)	0/2 (0%)
	HbA1c	6/12 (50%)	6/12 (50%)	0/11 (0%)
	Fasting plasma glucose	0/3 (0%)	3/3 (100%)	0/3 (0%)
	SBP	4/10 (40%)	6/10 (60%)	0/10 (0%)
	DBP	3/10 (30%)	7/10 (70%)	0/10 (0%)
	LDL	4/5 (80%)	1/5 (20%)	0/5 (0%)
	HDL	0/3 (0%)	3/3 (100%)	0/3 (0%)
	Total cholesterol	2/4 (50%)	2/4 (50%)	0/4 (0%)
	Triglycerides	1/4 (25%)	3/4 (75%)	0/4 (0%)
	10 year framingham risk score	0/2 (0%)	2/2 (100%)	0/2 (0%)
	BMI	2/5 (40%)	3/5 (60%)	0/5 (0%)
	Weight	2/5 (40%)	3/5 (60%)	0/5 (0%)
	Quality of life	10/17 (59%)	7/17 (41%)	0/17 (0%)
	Mortality	1/4 (25%)	3/4 (75%)	0/4 (0%)
Resource use	ED visits	3/8 (38%)	5/8 (63%)	0/7 (0%)
	Hospital admission/readmissions	4/18 (22%)	14/18 (78%)	0/18 (0%)
	Hospital admission: timing	0/3 (0%)	3/3 (100%)	0/3 (0%)
	HCP visits	0/10 (0%)	10/10 (100%)	0/10 (0%)
Satisfaction with care	Satisfaction with care	4/6 (66%)	2/6 (33%)	0/6 (0%)

### 3.5 Patient self‐management

Three patient self‐management outcomes were identified: (a) self‐efficacy, (b) self‐care and (c) treatment adherence. Self‐efficacy was measured nine times in eight different studies. Four of the nine times (44%) the construct was measured, patient self‐efficacy was improved due to nurse‐led digital health support. In the remaining five of nine (55%) studies, there was no significant change observed between the two groups. In four of the nine (44%) instances where self‐care was measured it was elevated in the intervention group. Intervention types associated with positive outcomes were websites (2/4; 50%) and mobile applications (2/4; 50%) and either frequently involved clinical data being sent to a nurse for review and appropriate response (2/4; 50%). Treatment adherence was measured 10 times across nine studies and was improved in the intervention group half the time (5/10). No intervention had a significantly negative impact on any self‐management outcome.

### 3.6 Clinical variables

#### 3.6.1 Mental health

Depression was measured nine times across nine studies and nurse‐led remote digital support improved depressions scores 78% of the time (7/9). In these seven studies, four interventions involved mobile applications and five involved nurses and patients interacting at least weekly. Depressive symptoms and their intensity were measured in two studies but did not differ between study groups. Anxiety was measured in two studies; both of which showed a nurse‐led intervention to improve patient anxiety.

#### 3.6.2 Diabetes‐related health

Haemoglobin A1C (HbA1c) levels were measured and compared in 12 studies and levels in the intervention group were significantly improved in 50% of the studies compared to control (6/12). Fifty per cent of the interventions (3/6) which significantly improved HbA1c involved at least biweekly contact between the nurse and patient. Fasting plasma glucose levels were measured three times without a between‐group change.

#### 3.6.3 Cardiovascular health

Systolic and diastolic blood pressure (SBP and DBP) were both measured 10 times in 10 different studies. SBP improved in four of 10 (40%) studies and DBP improved in three of 10 (30%). Low‐density lipoprotein (LDL) was measured five times and improved in four of five (80%) studies related to nurse‐led digital support while high‐density lipoprotein (HDL) levels did not differ between groups in the three studies which measured the blood marker (0/3). Total cholesterol and triglyceride levels improved due to nurse‐led intervention 50% of the time (2/4; 50%) and one of four (25%) respectively. Body mass index (BMI) and weight were both measured as potential risk factors for cardiovascular disease (CVD) in five studies and both improved related to nurse‐led support two of five (40%) times assessed. Cumulatively, overall risk for CVD was measured using the 10‐year Framingham Risk score in two studies; however no between group changes were observed.

#### 3.6.4 Additional health outcomes

Mortality was measured in four instances and was lowered in the nurse‐led intervention group 25% of the time. Quality of life (QoL) was assessed 17 times across 12 different studies. Fifty‐nine per cent of the time (10/17), nurse‐led digital remote support significantly improved participant QoL compared to usual care. In no studies did nurse‐led digital interventions negatively impact any aspect of participant clinical status.

### 3.7 Health care resource usage and satisfaction with care

Hospital admissions/readmissions outcomes were assessed 18 times across nine studies and decreased due to nurse intervention in four (4/18;22%). Emergency department visits were measured in eight studies and decreased in three (3/8; 38%) studies in the nurse‐led intervention group. Visits to a health care professional and time between hospital admissions were reported 10 and three times respectively without any between‐group changes observed. Satisfaction with care was assessed in six studies and significantly improved in the nurse group 66% of the time (4/6).

### 3.8 Impact of digital modality type

Table 3 shows outcome results per digital modality. Mobile applications were the most effective type of digital modality; significantly improving 21 of the 33 (64%) outcomes measured in associated studies. Nurse emails and nurse‐developed websites improved 50% (5/10) and 47% (16/34) of outcomes in their respective studies compared to usual care. This was followed by multicomponent interventions (6/18; 33%), novel monitoring systems (12/38; 32%) and texting interventions (4/13; 30%).

**TABLE 3:** Trends towards significant differences per digital modality.

Digital modality	Number of papers utilizing modality	Number of outcomes measured where modality utilized	Significantly positive outcome impact of nurse‐led remote digital support (*n*, %)
Mobile applications	10	33	21/33 (64)
Emails	2	10	5/10 (50)
Websites	10	34	16/34 (47)
Multi‐component systems	3	18	6/18 (33)
Novel monitoring systems	9	38	12/38 (32)
Text‐messaging	4	13	4/13 (30)
Video‐based platforms	2	7	1/7 (14)

### 3.9 Risk of bias‐assessment

**FIGURE 3:**
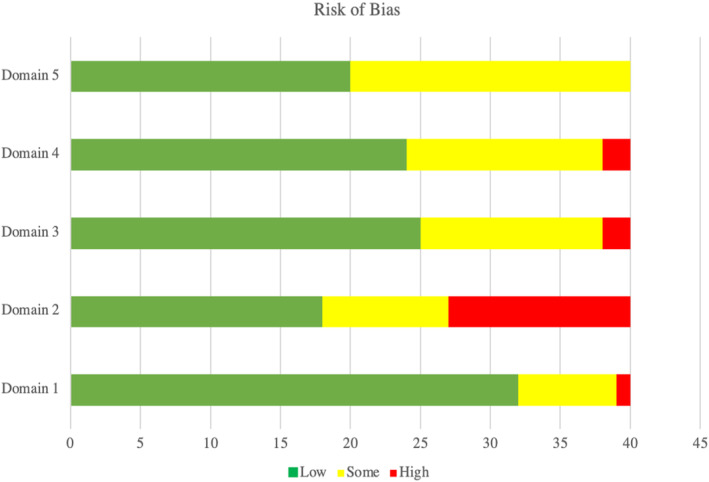
Risk of bias assessment. Overall summary for included randomized controlled trials assessed using the Cochrane collaboration tool. [Colour figure can be viewed at wileyonlinelibrary.com]

Twenty‐four (60%) studies were rated as having high risk of bias, 12 (30%) as ‘some concerns’, and four (10%) as ‘low concern’ (Table 4). The bias domain with the highest risk across studies was deviation from intended interventions (13/40; 33%) (Figure 3). The randomization process was the domain with the lowest risk of bias (32/40; 80%) (Figure 3).

**TABLE 4:** Risk of bias assessment results. [Colour table can be viewed at wileyonlinelibrary.com]

Study	Risk of bias domains					
	Domain 1	Domain 2	Domain 3	Domain 4	Domain 5	Overall
Ahmed et al., 2016	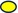	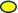	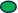	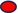	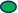	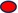
Arad et al., 2021	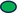	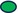	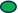	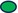	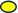	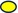
Bell et al., 2012	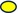	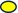	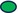	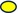	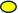	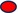
Chen et al., 2022	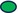	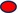	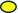	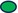	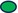	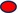
Cicolini et al., 2014	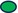	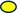	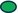	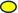	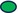	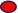
Egede et al., 2017; Egede et al., 2021	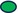	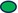	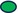	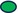	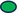	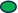
Greving et al., 2015; Vernooij et al., 2012	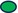	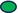	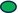	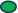	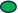	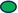
Jiang et al., 2021	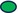	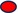	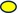	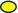	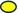	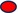
Jiang et al., 2022	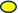	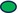	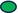	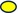	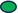	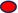
Kenealy et al., 2015	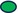	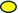	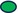	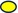	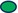	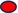
Kes & Polat, 2022	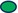	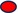	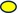	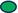	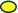	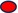
Kim, 2007; Kim & Jeong, 2007	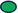	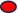	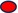	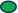	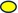	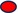
Lyu et al., 2021	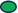	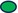	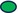	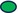	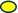	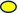
Liang et al., 2021	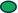	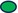	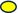	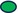	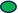	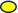
Metilda et al., 2021	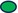	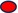	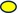	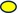	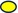	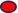
Mir et al., 2022	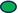	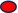	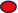	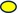	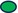	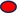
Mumcu et al., 2022	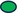	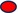	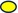	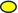	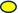	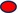
Pakrad et al., 2021	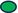	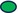	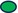	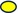	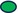	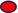
Parker et al., 2022	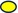	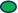	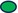	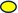	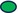	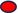
Ritchie et al., 2016	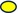	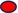	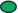	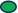	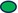	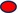
Sawyer et al., 2019	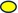	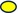	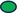	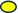	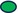	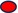
Shea et al., 2006; Shea et al., 2009	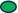	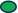	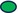	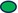	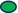	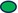
Simon et al., 2011	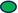	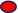	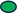	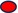	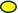	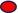
Simsek‐Cetinkaya & Koc, 2022	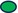	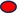	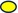	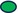	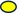	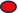
Sorknaes et al., 2013	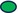	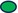	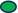	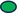	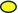	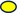
Southard et al., 2003	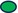	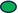	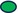	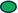	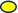	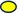
Su & Yu, 2021	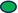	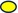	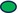	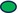	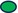	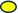
Su et al., 2021	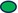	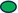	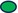	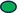	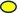	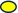
Tang et al., 2013	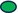	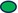	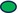	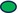	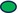	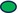
Tonning et al., 2021	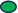	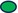	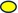	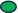	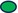	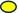
Valdivieso et al., 2018	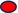	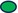	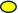	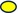	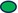	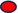
Wakefield et al., 2009	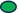	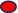	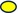	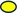	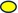	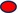
Wakefield et al., 2011	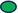	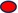	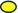	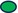	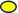	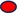
Wakefield et al., 2014	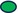	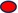	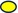	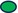	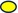	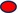
Wang et al., 2018	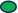	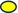	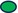	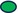	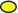	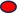
Wheelock et al., 2015	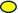	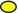	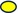	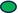	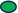	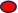
Willems et al., 2008	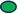	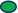	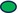	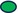	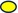	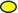
Wong et al., 2022	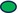	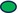	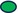	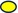	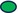	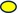
Yan et al., 2022	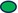	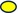	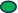	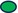	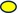	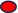
Zhang et al., 2018	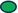	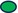	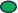	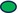	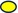	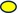

*Note*: Domains: D1: Bias arising from the randomization process, D2: Bias due to deviations from intended intervention, D3: Bias due to missing outcome data, D4: Bias in measurement of the outcome, D5: Bias in selection of the reported result. 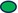
Low risk, 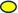
Some concerns, 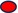
High risk.

## 4 DISCUSSION

The impact of nurse‐led remote digital management on health outcomes in adults with chronic conditions was investigated. Forty studies with a cumulative positive effect on observer‐ and patient‐reported outcomes were identified. Digital modalities evaluated through studies were most commonly websites and mobile applications followed by novel monitoring systems, text messaging, emails, and video‐based platforms. Nurses provided support to participants either in response to receiving clinical data, at virtual appointments, through virtual health education or through a combination of these means. Nurse‐led digital management significantly improved patient‐self management outcomes, specifically self‐efficacy, self‐care and treatment adherence, clinical variables related to mental health, diabetes and cardiovascular health, health care resource usage and satisfaction with care.

Recognizing evidence from this review indicating that nurse‐led digital health interventions may positively impact health outcomes broadly, questions about the contextual influences on effectiveness arise. For instance, identified studies were published between 2003 and 2022 and most commonly conducted in high‐income countries or contexts. This highlights the ongoing digital health divide whereby research in the area is infrequently conducted and interventions are rarely implemented in settings lacking available funding, technological infrastructure, and access to skilled nurses trained in both technology and specific chronic disease care (Lyles et al., 2021). Interventions identified in our review also generally targeted a younger population of adults, with only a handful of studies focused on adult patients older than 70 years. This exclusion of older adults in technology‐based research has been previously noted before and may reflect clinician and researcher misconceptions around the technological literacy of older adults (Chu et al., 2022; Mannheim et al., 2019). However, the strong evidence that older adults both use and report benefiting from technology (Chu et al., 2022) coupled with the prevalence of chronic conditions within the older adult group, future nurse‐engaged interventional research within this population is needed.

Nurse‐led digital health interventions significantly improved subjectively reported clinical outcomes, including depression, anxiety and quality of life compared to usual care. Other systematic reviews have highlighted the ability of digital‐remote monitoring to improve patient‐reported health, but this review is the first to demonstrate the impact of nurse‐led interventions in this regard (McLean et al., 2016). Nurse‐led digital support also significantly improved patient‐self management and patient satisfaction with care, reinforcing findings that show patients desire means to manage illness safely in their homes (Kim et al., 2017). Overall, however, nurse‐led interventions had less impact on objectively measured clinical outcomes including blood triglyceride level and body mass index and health care resource‐related outcomes health care professional visit number. Longer follow‐up periods may be needed to observe changes in these outcomes, which may sit downstream from those related to increasing patient capacity to self‐manage their chronic conditions at home.

Future studies should compare the effect of nurse‐led digital‐monitoring with digital‐monitoring without a nurse to further parse out the role of nurse care in remote chronic disease support. Considering how nurse engagement might influence digital intervention effectiveness, our review points to a potential necessity for relatively frequent nurse–patient contact. Studies where nurses interacted with patients at least weekly more frequently reported positive results in mental health outcomes. This supports previous work showing that patients with mental health conditions benefit from or desire a steady schedule of clinician involvement in their care (Goodwin et al., 2016; Jibb et al., 2018, 2023). Interventions delivered through mobile applications more frequently reported positive results. While included interventions were not consistently described in great detail, this finding may relate to the capacity for mobile applications to offer engaging features (animations, interactive components), multiple forms of support (disease and care education, online support forums, nurse chats) and enhanced accessibility to care in nearly all environments. These factors have been previously identified in the literature as important in developing an engaging and effective digital health intervention (Dawson et al., 2020).

## 5 IMPLICATIONS FOR NURSING RESEARCH AND CLINICAL PRACTICE

Future research should evaluate the nursing characteristics necessary to further strengthen the interventions. Specifications related to clinical and technological education and experience is needed to understand how to best provide remote chronic disease support to patients. Evaluations of how best to integrate digital patient care into established nursing workflows are also necessary. Research should also focus on how nurses, other clinicians, patients and their families can actively participate in the design, development, evaluation and implementation of these interventions. The involvement of older adults throughout the development of digital support interventions is especially crucial, considering the aging population and their increased susceptibility to chronic conditions. Co‐design, a method strongly supported by the digital health community, serves as a powerful approach to promote adherence to and participation in successful interventions (Noorbergen et al., 2021).

Assessments of nurse fidelity to intervention delivery are required in nurse‐led remote digital disease support. Few identified studies assessed and accounted for fidelity in their analysis, which is problematic as the degree of participant adherence to digital health interventions largely impacts effectiveness (Jakob et al., 2022). In addition, contextual factors involving the participant's physical and economic environment, culture, socio‐demographics, including age sex, gender, race and ethnicity and technological literacy were left unaddressed. These factors underpin the digital divide in healthcare and are known to influence participant adherence, as well as intervention usage and satisfaction, and must be accounted for while designing, implementing and evaluating digital health technologies (Amagai et al., 2022). Several identified studies were rated as having high risk of bias due to a lack of participant blinding and a high rate of participant attrition. Although challenging to blind participants without an active control group, future studies should aim to blind outcome assessors and analysts to minimize risk of bias. Engagement strategies including push notifications and gamification should also be incorporated to decrease participant attrition (Jakob et al., 2022). Finally, the results of this review may represent publication bias in included studies whereby studies reporting positive intervention findings are more likely to be published.

## 6 LIMITATIONS

Several limitations of this review should be highlighted. The search was only limited to English language studies, and relevant studies published in other languages may have been excluded. In addition, due to the heterogeneity of included studies, a meta‐analysis to explore an overall effect of nurse‐led digital health interventions for chronic disease was not possible. Furthermore, only randomized controlled trials comparing the effectiveness of nurse‐led interventions to usual medical care were included, limiting the capacity to discuss intervention effectiveness—including through qualitative reports with patient participants—in a holistic way.

## 7 CONCLUSION

In conclusion, this systematic review is the first to report on the impact that nurse‐led digital remote support can have on adult patients with chronic illness. Taken together, the review offers evidence of the positive effect such interventions may have on patient self‐management capacity and its downstream targets of clinical health, system resource use and care satisfaction. Through this process several areas of research to be expanded on were identified including examining the role of nurses in these interventions, assessing the impact of socio‐economic and resource availability on intervention use and including participant fidelity as a confounding variable in future studies. Nurses are health care professionals who are experts in supporting patients with chronic diseases in a clinical setting (Griffin, 2017). Given this expertise, the results of this study, and the increasing affordability of digital technologies, nurse‐led digital remote management is well‐positioned to support the quality of care and quality of life of patients with chronic illness in all environments, including their own homes.

## AUTHOR CONTRIBUTIONS

Lindsay Jibb and Charlene Chu conceptualized the review idea, design and search strategy and provided supervision. Alicia Kilfoy, Archanaa Krisnagopal, Enoch McAtee, Sunny Baek, Mallory Zworth, Kyobin Hwang and Huyn Park contributed to the investigator role through data collection. Alicia Kilfoy contributed to the project administration, data visualization and validation roles. The initial manuscript draft was written by Alicia Kilfoy and Lindsay Jibb. All authors critically reviewed the first draft and approved prior to publication.

## FUNDING INFORMATION

None.

## CONFLICT OF INTEREST STATEMENT

Nothing to declare.

## TWITTER

Alicia Kilfoy: @akilfoy

Charlene Chu: @CharchuRn

Sunny Baek: @SunnyBae_K

Lindsay Jibb: @lindsayjibb

Lawrence S. Bloomberg Faculty of Nursing: @UofTNursing

The Hospital for Sick Children: @SickKidsNews

KITE Research Institute: @KITE_UHN

## Supporting information


Appendix S1.

Appendix S2.

## Data Availability

The data that support the findings of this study are available from the corresponding author upon reasonable request.

## References

[jocn17226-bib-0001] Ahmed, S. , Ernst, P. , Bartlett, S. J. , Valois, M.‐F. , Zaihra, T. , Paré, G. , Grad, R. , Eilayyan, O. , Perreault, R. , & Tamblyn, R. (2016). The effectiveness of web‐based asthma self‐management system, my asthma portal (MAP): A pilot randomized controlled trial. Journal of Medical Internet Research, 18(12), e313. 10.2196/jmir.5866 27908846 PMC5159614

[jocn17226-bib-0002] Amagai, S. , Pila, S. , Kaat, A. J. , Nowinski, C. J. , & Gershon, R. C. (2022). Challenges in participant engagement and retention using mobile health apps: Literature review. Journal of Medical Internet Research, 24(4), e35120. 10.2196/35120 35471414 PMC9092233

[jocn17226-bib-0003] Ansah, J. P. , & Chiu, C. T. (2023). Projecting the chronic disease burden among the adult population in the United States using a multi‐state population model. Frontiers in Public Health, 10, 1082183. 10.3389/fpubh.2022.1082183 36711415 PMC9881650

[jocn17226-bib-0004] Antes, A. L. , Burrous, S. , Sisk, B. A. , Schuelke, M. J. , Keune, J. D. , & DuBois, J. M. (2021). Exploring perceptions of healthcare technologies enabled by artificial intelligence: An online, scenario‐based survey. BMC Medical Informatics and Decision Making, 21(1), 221. 10.1186/s12911-021-01586-8 34284756 PMC8293482

[jocn17226-bib-0005] Arad, M. , Goli, R. , Parizad, N. , Vahabzadeh, D. , & Baghaei, R. (2021). Do the patient education program and nurse‐led telephone follow‐up improve treatment adherence in hemodialysis patients? A randomized controlled trial. BMC Nephrology, 22(1), 1–13. 10.1186/s12882-021-02319-9 33827478 PMC8028152

[jocn17226-bib-0006] Bell, A. M. , Fonda, S. J. , Walker, M. S. , Schmidt, V. , & Vigersky, R. A. (2012). Mobile phone‐based video messages for diabetes self‐care support. Journal of Diabetes Science and Technology, 6(2), 310–319. 10.1177/193229681200600214 22538140 PMC3380772

[jocn17226-bib-0007] Booth, R. G. , Strudwick, G. , McBride, S. , O'Connor, S. , & Solano López, A. L. (2021). How the nursing profession should adapt for a digital future. The BMJ, 373, n1190. 10.1136/bmj.n1190

[jocn17226-bib-0008] Campbell, M. , McKenzie, J. E. , Sowden, A. , Katikireddi, S. V. , Brennan, S. E. , Ellis, S. , Hartmann‐Boyce, J. , Ryan, R. , Shepperd, S. , Thomas, J. , Welch, V. , & Thomson, H. (2020). Synthesis without meta‐analysis (SWiM) in systematic reviews: Reporting guideline. BMJ, 368, l6890. 10.1136/bmj.l6890 31948937 PMC7190266

[jocn17226-bib-0009] Chen, H. H. , Lee, C. F. , Huang, J. P. , Hsiung, Y. , & Chi, L. K. (2022). Effectiveness of a nurse‐led mHealth app to prevent excessive gestational weight gain among overweight and obese women: A randomized controlled trial. Journal of Nursing Scholarship: An Official Publication of Sigma Theta Tau International Honor Society of Nursing., 55, 304–318. 10.1111/jnu.12813 36121127

[jocn17226-bib-0010] Chu, C. H. , Nyrup, R. , Leslie, K. , Shi, J. , Bianchi, A. , Lyn, A. , McNicholl, M. , Khan, S. , Rahimi, S. , & Grenier, A. (2022). Digital ageism: Challenges and opportunities in artificial intelligence for older adults. The Gerontologist, 62(7), 947–955. 10.1093/geront/gnab167 35048111 PMC9372891

[jocn17226-bib-0011] Cicolini, G. , Simonetti, V. , Comparcini, D. , Celiberti, I. , Di Nicola, M. , Capasso, L. M. , Flacco, M. E. , Bucci, M. , Mezzetti, A. , & Manzoli, L. (2014). Efficacy of a nurse‐led email reminder program for cardiovascular prevention risk reduction in hypertensive patients: A randomized controlled trial. International Journal of Nursing Studies, 51(6), 833–843. 10.1016/j.ijnurstu.2013.10.010 24225325

[jocn17226-bib-0012] Dawson, R. M. , Felder, T. M. , Donevant, S. B. , McDonnell, K. K. , Card, E. B. , King, C. C. , & Heiney, S. P. (2020). What makes a good health ‘app’? Identifying the strengths and limitations of existing mobile application evaluation tools. Nursing Inquiry, 27(2), e12333. 10.1111/nin.12333 31854055 PMC7181198

[jocn17226-bib-0013] Egede, L. E. , Dawson, A. Z. , Walker, R. J. , Garraci, E. , & Knapp, R. G. (2021). Randomized controlled trial of technology‐assisted case management in low‐income adults with type 2 diabetes: Effect on quality of life and blood pressure. Journal of Telemedicine and Telecare, 30(1), 107–115. 10.1177/1357633X211028491 34251865

[jocn17226-bib-0014] Egede, L. E. , Walker, R. J. , Garacci, E. , & Williams, J. S. (2020). *Randomized controlled trial of technology‐assisted case management in low‐income adults with type 2 diabetes (TACMDM): Effect on quality of life and blood pressure*. *69* . 10.1177/1357633X211028491 34251865

[jocn17226-bib-0015] Golics, C. J. , Basra, M. , Salek, M. S. , & Finlay, A. Y. (2013). The impact of patients' chronic disease on family quality of life: An experience from 26 specialties. International Journal of General Medicine, 6, 787–798. 10.2147/IJGM.S45156 24092994 PMC3787893

[jocn17226-bib-0016] Goodwin, J. , Cummins, J. , Behan, L. , & O'Brien, S. M. (2016). Development of a mental health smartphone app: Perspectives of mental health service users. Journal of Mental Health, 25(5), 434–440. 10.3109/09638237.2015.1124392 26732242

[jocn17226-bib-0017] Greving, J. P. , Kaasjager, H. A. H. , Vernooij, J. W. P. , Hovens, M. M. C. , Wierdsma, J. , Grandjean, H. M. H. , Van Der Graaf, Y. , De Wit, G. A. , & Visseren, F. L. J. (2015). Cost‐effectiveness of a nurse‐led internet‐based vascular risk factor management programme: Economic evaluation alongside a randomised controlled clinical trial. BMJ Open, 5(5), e007128. 10.1136/bmjopen-2014-007128 PMC4442232 25995238

[jocn17226-bib-0018] Griffin, C. D. (2017). A primary care nursing perspective on chronic disease prevention and management. Delaware Journal of Public Health, 3(1), 78–83. 10.32481/djph.2017.03.011 34466901 PMC8352460

[jocn17226-bib-0019] Hanley, J. , Fairbrother, P. , McCloughan, L. , Pagliari, C. , Paterson, M. , Pinnock, H. , Sheikh, A. , Wild, S. , & McKinstry, B. (2015). Qualitative study of telemonitoring of blood glucose and blood pressure in type 2 diabetes. BMJ Open, 5(12), e008896. 10.1136/bmjopen-2015-008896 PMC4691739 26700275

[jocn17226-bib-0020] Jakob, R. , Harperink, S. , Rudolf, A. M. , Fleisch, E. , Haug, S. , Mair, J. L. , Salamanca‐Sanabria, A. , & Kowatsch, T. (2022). Factors influencing adherence to mHealth apps for prevention or management of noncommunicable diseases: Systematic review. Journal of Medical Internet Research, 24(5), e35371. 10.2196/35371 35612886 PMC9178451

[jocn17226-bib-0021] Jeon, Y. H. , Essue, B. , Jan, S. , Wells, R. , & Whitworth, J. A. (2009). Economic hardship associated with managing chronic illness: A qualitative inquiry. BMC Health Services Research, 9(1), 182. 10.1186/1472-6963-9-182 19818128 PMC2766369

[jocn17226-bib-0022] Jiang, Y. , Koh, K. W. L. , Ramachandran, H. J. , Nguyen, H. D. , Lim, S. , Tay, Y. K. , Shorey, S. , & Wang, W. (2021). The effectiveness of a nurse‐led home‐based heart failure self‐management programme (the HOM‐HEMP) for patients with chronic heart failure: A three‐arm stratified randomized controlled trial. International Journal of Nursing Studies, 122, 104026. 10.1016/j.ijnurstu.2021.104026 34271265

[jocn17226-bib-0023] Jiang, Y. , Ramachandran, H. J. , Teo, J. Y. C. , Leong, F. L. , Lim, S. T. , Nguyen, H. D. , & Wang, W. (2022). Effectiveness of a nurse‐led smartphone‐based self‐management programme for people with poorly controlled type 2 diabetes: A randomized controlled trial. Journal of Advanced Nursing (John Wiley & Sons, Inc.), 78(4), 1154–1165. 10.1111/jan.15178 35170786

[jocn17226-bib-0024] Jibb, L. A. , Chartrand, J. , Masama, T. , & Johnston, D. L. (2021). Home‐based pediatric cancer care: Perspectives and improvement suggestions from children, family caregivers, and clinicians. JCO Oncology Practice, 17(6), e827–e839. 10.1200/OP.20.00958 33914620

[jocn17226-bib-0025] Jibb, L. A. , Sivaratnam, S. , Hashemi, E. , Chu, C. H. , Nathan, P. C. , Chartrand, J. , Alberts, N. M. , Masama, T. , Pease, H. G. , Torres, L. B. , Cortes, H. G. , Zworth, M. , Kuczynski, S. , & Fortier MA . (2023). Parent and clinician perceptions and recommendations on a pediatric cancer pain management app: a qualitative co‐design study. PLOS Digital Health, 2, e0000169. 38019890 10.1371/journal.pdig.0000169 PMC10686487

[jocn17226-bib-0026] Jibb, L. A. , Stevens, B. J. , Nathan, P. C. , Seto, E. , Cafazzo, J. A. , Johnston, D. L. , Hum, V. , & Stinson, J. N. (2018). Perceptions of adolescents with cancer related to a pain management app and its evaluation: Qualitative study nested within a multicenter pilot feasibility study. JMIR mHealth and uHealth, 6(4), e80. 10.2196/mhealth.9319 29625951 PMC5910537

[jocn17226-bib-0027] Kenealy, T. W. , Parsons, M. J. G. , Rouse, A. P. B. , Doughty, R. N. , Sheridan, N. F. , Hindmarsh, J. K. H. , Masson, S. C. , & Rea, H. H. (2015). Telecare for diabetes, CHF or COPD: Effect on quality of life, hospital use and costs. A randomised controlled trial and qualitative evaluation. PLoS One, 10(3), e0116188. 10.1371/journal.pone.0116188 25768023 PMC4358961

[jocn17226-bib-0028] Kes, D. , & Polat, U. (2022). The effect of nurse‐led telephone support on adherence to blood pressure control and drug treatment in individuals with primary hypertension: A randomized controlled study. International Journal of Nursing Practice (John Wiley & Sons, Inc.), 28(3), 1–10. 10.1111/ijn.12995 34318542

[jocn17226-bib-0029] Kim, H. (2007). Impact of web‐based nurse's education on glycosylated haemoglobin in type 2 diabetic patients. Journal of Clinical Nursing, 16, 1361–1366. 10.1111/j.1365-2702.2005.01506.x 17584355

[jocn17226-bib-0030] Kim, H. , & Jeong, H. (2007). A nurse short message service by cellular phone in type‐2 diabetic patients for six months. Journal of Clinical Nursing, 16(6), 1082–1087. 10.1111/j.1365-2702.2007.01698.x 17518883

[jocn17226-bib-0031] Kim, K. I. , Gollamudi, S. S. , & Steinhubl, S. (2017). Digital technology to enable aging in place. Experimental Gerontology, 88, 25–31. 10.1016/j.exger.2016.11.013 28025126

[jocn17226-bib-0032] Liang, H. Y. , Hann Lin, L. , Yu Chang, C. , Mei, W. , & Yu, S. (2021). Effectiveness of a nurse‐led tele‐homecare program for patients with multiple chronic illnesses and a high risk for readmission: A randomized controlled trial. Journal of Nursing Scholarship, 53(2), 161–170. 10.1111/jnu.12622 33507626

[jocn17226-bib-0033] Lyles, C. R. , Wachter, R. M. , & Sarkar, U. (2021). Focusing on digital health equity. JAMA, 326(18), 1795. 10.1001/jama.2021.18459 34677577

[jocn17226-bib-0034] Lyu, Q. , Huang, J. , Li, Y. , Chen, Q. , Yu, X. , Wang, J. , & Yang, Q. (2021). Effects of a nurse led web‐based transitional care program on the glycemic control and quality of life post hospital discharge in patients with type 2 diabetes: A randomized controlled trial. International Journal of Nursing Studies, 119, 103929. 10.1016/j.ijnurstu.2021.103929 33901941

[jocn17226-bib-0035] Madanian, S. , Nakarada‐Kordic, I. , Reay, S. , & Chetty, T. (2023). Patients' perspectives on digital health tools. PEC Innovation., 2, 100171. 10.1016/j.pecinn.2023.100171 37384154 PMC10294099

[jocn17226-bib-0036] Mannheim, I. , Schwartz, E. , Xi, W. , Buttigieg, S. C. , McDonnell‐Naughton, M. , Wouters, E. J. , & Van Zaalen, Y. (2019). Inclusion of older adults in the research and design of digital technology. International Journal of Environmental Research and Public Health, 16(19), 3718. 31581632 10.3390/ijerph16193718 PMC6801827

[jocn17226-bib-0037] McGuinness, L. A. , & Higgins, J. P. T. (2020). Risk‐of‐bias VISualization (robvis): An R package and shiny web app for visualizing risk‐of‐bias assessments. Research Synthesis Methods, 12, 55–61. 10.1002/jrsm.1411 32336025

[jocn17226-bib-0038] McLean, G. , Band, R. , Saunderson, K. , Hanlon, P. , Murray, E. , Little, P. , McManus, R. J. , Yardley, L. , & Mair, F. S. (2016). Digital interventions to promote self‐management in adults with hypertension systematic review and meta‐analysis. Journal of Hypertension, 34(4), 600–612. 10.1097/HJH.0000000000000859 26845284 PMC4947544

[jocn17226-bib-0039] McLean, S. , Nurmatov, U. , Liu, J. L. , Pagliari, C. , Car, J. , & Sheikh, A. (2012). Telehealthcare for chronic obstructive pulmonary disease: Cochrane review and meta‐analysis. The British Journal of General Practice, 62(604), e739–e749. 10.3399/bjgp12X658269 23211177 PMC3481514

[jocn17226-bib-0040] Metilda, C. J. , Sharma, K. K. , Sinha, A. P. , & Agrawal, D. (2021). Effectiveness of nurse‐driven discharge teaching using mobile application for home‐based health care practices among postoperative neurosurgical patients or caregivers in a tertiary care hospital, New Delhi: A randomized control study. Indian Journal of Neurotrauma, (Metilda), 18(2), 119–125. 10.1055/s-0041-1724143

[jocn17226-bib-0041] Mir, O. , Ferrua, M. , Fourcade, A. , Mathivon, D. , Duflot‐Boukobza, A. , Dumont, S. , Baudin, E. , Delaloge, S. , Malka, D. , Albiges, L. , Pautier, P. , Robert, C. , Planchard, D. , de Botton, S. , Scotte, F. , Lemare, F. , Abbas, M. , Guillet, M. , Puglisi, V. , & Minvielle, E. (2022). Digital remote monitoring plus usual care versus usual care in patients treated with oral anticancer agents: The randomized phase 3 CAPRI trial. Nature Medicine, 28(6), 1224–1231. 10.1038/s41591-022-01788-1 35469070

[jocn17226-bib-0042] Mumcu, C. D. , & Vardar İnkaya, B. (2022). Investigation of the effect of web‐based education on self‐care management and family support in women with type 2 diabetes. The Journal for Nurse Practitioners, 18(8), 867–871. 10.1016/j.nurpra.2022.05.018

[jocn17226-bib-0043] Nakamura, N. , Koga, T. , & Iseki, H. (2014). A meta‐analysis of remote patient monitoring for chronic heart failure patients. Journal of Telemedicine and Telecare, 20(1), 11–17. 10.1177/1357633X13517352 24352899

[jocn17226-bib-0044] Noorbergen, T. J. , Adam, M. T. P. , Teubner, T. , & Collins, C. E. (2021). Using co‐design in mobile health system development: A qualitative study with experts in co‐design and mobile health system development. JMIR mHealth and uHealth, 9(11), e27896. 10.2196/27896 34757323 PMC8663505

[jocn17226-bib-0045] Pakrad, F. , Ahmadi, F. , Grace, S. L. , Oshvandi, K. , & Kazemnejad, A. (2021). Traditional vs extended hybrid cardiac rehabilitation based on the continuous care model for patients who have undergone coronary artery bypass surgery in a middle‐income country: A randomized controlled trial. Archives of Physical Medicine and Rehabilitation, 102(11), 2091–2101.e3. 10.1016/j.apmr.2021.04.026 34175270

[jocn17226-bib-0046] Parker, S. M. , Barr, M. , Stocks, N. , Denney‐Wilson, E. , Zwar, N. , Karnon, J. , Kabir, A. , Nutbeam, D. , Roseleur, J. , Liaw, S.‐T. , McNamara, C. , Frank, O. , Tran, A. , Osborne, R. , Lau, A. Y. S. , & Harris, M. (2022). Preventing chronic disease in overweight and obese patients with low health literacy using eHealth and teamwork in primary healthcare (HeLP‐GP): A cluster randomised controlled trial. BMJ Open, 12(11), e060393. 10.1136/bmjopen-2021-060393 PMC9716831 36450426

[jocn17226-bib-0047] Ritchie, C. S. , Houston, T. K. , Richman, J. S. , Sobko, H. J. , Berner, E. S. , Taylor, B. B. , Salanitro, A. H. , & Locher, J. L. (2016). The E‐coach technology‐assisted care transition system: A pragmatic randomized trial. Translational Behavioral Medicine, 6(3), 428–437. 10.1007/s13142-016-0422-8 27339715 PMC4987612

[jocn17226-bib-0048] Sawyer, A. , Kaim, A. , Le, H.‐N. , McDonald, D. , Mittinty, M. , Lynch, J. , & Sawyer, M. (2019). The effectiveness of an app‐based nurse‐moderated program for new mothers with depression and parenting problems (eMums plus): Pragmatic randomized controlled trial. Journal of Medical Internet Research, 21(6), e13689. 10.2196/13689 31165715 PMC6682297

[jocn17226-bib-0049] Shea, S. , Weinstock, R. S. , Starren, J. , Teresi, J. , Palmas, W. , Field, L. , Morin, P. , Goland, R. , Izquierdo, R. E. , Wolff, L. T. , Ashraf, M. , Hilliman, C. , Silver, S. , Meyer, S. , Holmes, D. , Petkova, E. , Capps, L. , Lantigua, R. A. , & For the IDEATel Consortium . (2006). A randomized trial comparing telemedicine case management with usual care in older, ethnically diverse, medically underserved patients with diabetes mellitus. Journal of the American Medical Informatics Association, 13(1), 40–51. 10.1197/jamia.M1917 16221935 PMC1380195

[jocn17226-bib-0050] Shea, S. , Weinstock, R. S. , Teresi, J. A. , Palmas, W. , Starren, J. , Cimino, J. J. , Lai, A. M. , Field, L. , Morin, P. C. , Goland, R. , Izquierdo, R. E. , Ebner, S. , Silver, S. , Petkova, E. , Kong, J. , Eimicke, J. P. , Shea, S. , Weinstock, R. , Teresi, J. A. , & Palmas, W. (2009). A randomized trial comparing telemedicine case management with usual care in older, ethnically diverse, medically underserved patients with diabetes mellitus: 5 year results of the IDEATel study. Journal of the American Medical Informatics Association, 16(4), 446–456. 10.1197/jamia.M3157 19390093 PMC2705246

[jocn17226-bib-0051] Simon, G. E. , Ralston, J. D. , Savarino, J. , Pabiniak, C. , Wentzel, C. , & Operskalski, B. H. (2011). Randomized trial of depression follow‐up care by online messaging. Journal of General Internal Medicine, 26(7), 698–704. 10.1007/s11606-011-1679-8 21384219 PMC3138593

[jocn17226-bib-0052] Simsek‐Cetinkaya, S. , & Koc, G. (2022). Effects of a smartphone‐based nursing counseling and feedback system for women with gestational diabetes on compliance, glycemic control, and satisfaction: A randomized controlled study. International Journal of Diabetes in Developing Countries, 43, 529–537. 10.1007/s13410-022-01142-8

[jocn17226-bib-0053] Sorknaes, A. D. , Bech, M. , Madsen, H. , Titlestad, I. L. , Hounsgaard, L. , Hansen‐Nord, M. , Jest, P. , Olesen, F. , Lauridsen, J. , & Ostergaard, B. (2013). The effect of real‐time teleconsultations between hospital‐based nurses and patients with severe COPD discharged after an exacerbation. Journal of Telemedicine and Telecare, 19(8), 466–474. 10.1177/1357633X13512067 24227799

[jocn17226-bib-0054] Southard, B. H. , Southard, D. R. , & Nuckolls, J. (2003). Clinical trial of an internet‐based case management system for secondary prevention of heart disease. Journal of Cardiopulmonary Rehabilitation, 23(5), 341–348. 14512778 10.1097/00008483-200309000-00003

[jocn17226-bib-0055] Steffler, M. , Li, Y. , Weir, S. , Shaikh, S. , Murtada, F. , Wright, J. G. , & Kantarevic, J. (2021). Trends in prevalence of chronic disease and multimorbidity in Ontario, Canada. Canadian Medical Association Journal, 193(8), E270–E277. 10.1503/cmaj.201473 33619067 PMC8034347

[jocn17226-bib-0056] Sterne, J. A. C. , Savović, J. , Page, M. J. , Elbers, R. G. , Blencowe, N. S. , Boutron, I. , Cates, C. J. , Cheng, H.‐Y. , Corbett, M. S. , Eldridge, S. M. , Emberson, J. R. , Hernán, M. A. , Hopewell, S. , Hróbjartsson, A. , Junqueira, D. R. , Jüni, P. , Kirkham, J. J. , Lasserson, T. , Li, T. , … Higgins, J. P. T. (2019). RoB 2: A revised tool for assessing risk of bias in randomised trials. BMJ, 366, l4898. 10.1136/bmj.l4898 31462531

[jocn17226-bib-0057] Su, J. J. , & Yu, D. S. (2021). Effects of a nurse‐led eHealth cardiac rehabilitation programme on health outcomes of patients with coronary heart disease: A randomised controlled trial. International Journal of Nursing Studies, 122, 104040. 10.1016/j.ijnurstu.2021.104040 34333211

[jocn17226-bib-0058] Su, M. C. , Chao, A. S. , Chang, M. Y. , Chang, Y. L. , Chen, C. L. , & Sun, J. C. (2021). Effectiveness of a nurse‐led web‐based health management in preventing women with gestational diabetes from developing metabolic syndrome. Journal of Nursing Research, 29(6), e176. 10.1097/jnr.0000000000000456 34570053

[jocn17226-bib-0059] Tang, P. C. , Overhage, J. M. , Chan, A. S. , Brown, N. L. , Aghighi, B. , Entwistle, M. P. , Hui, S. L. , Hyde, S. M. , Klieman, L. H. , Mitchell, C. J. , Perkins, A. J. , Qureshi, L. S. , Waltimyer, T. A. , Winters, L. J. , & Young, C. Y. (2013). Online disease management of diabetes: Engaging and motivating patients online with enhanced resources‐diabetes (EMPOWER‐D), a randomized controlled trial. Journal of the American Medical Informatics Association, 20(3), 526–534. 10.1136/amiajnl-2012-001263 23171659 PMC3628059

[jocn17226-bib-0060] The Dutch National Consensus Committee “Chronic Diseases and Health Conditions in Childhood” , Mokkink, L. B. , Van Der Lee, J. H. , Grootenhuis, M. A. , Offringa, M. , & Heymans, H. S. A. (2008). Defining chronic diseases and health conditions in childhood (0–18 years of age): National consensus in The Netherlands. European Journal of Pediatrics, 167(12), 1441–1447. 10.1007/s00431-008-0697-y 18340463

[jocn17226-bib-0061] Tonning, M. L. , Faurholt‐Jepsen, M. , Frost, M. , Martiny, K. , Tuxen, N. , Rosenberg, N. , Busk, J. , Winther, O. , Melbye, S. A. , Thaysen‐Petersen, D. , Aamund, K. A. , Tolderlundh, L. , Bardram, J. E. , & Kessing, L. V. (2021). The effect of smartphone‐based monitoring and treatment on the rate and duration of psychiatric readmission in patients with unipolar depressive disorder: The RADMIS randomized controlled trial. Journal of Affective Disorders, 282, 354–363. 10.1016/j.jad.2020.12.141 33421863

[jocn17226-bib-0062] Troncoso, E. L. , & Breads, J. (2021). Best of both worlds: Digital health and nursing together for healthier communities. International Nursing Review, 68(4), 504–511. 10.1111/inr.12685 34133028

[jocn17226-bib-0063] Ure, J. , Pinnock, H. , Hanley, J. , Kidd, G. , Smith, E. M. C. , Tarling, A. , Pagliari, C. , Sheikh, A. , MacNee, W. , & McKinstry, B. (2011). Piloting tele‐monitoring in COPD: A mixed methods exploration of issues in design and implementation. Primary Care Respiratory Journal, 21(1), 57–64. 10.4104/pcrj.2011.00065 PMC6548305 21785816

[jocn17226-bib-0064] Valdivieso, B. , García‐Sempere, A. , Sanfélix‐Gimeno, G. , Faubel, R. , Librero, J. , Soriano, E. , & Peiró, S. (2018). The effect of telehealth, telephone support or usual care on quality of life, mortality and healthcare utilization in elderly high‐risk patients with multiple chronic conditions. A prospective study. Medicina Clínica (English Edition), 151(8), 308–314. 10.1016/j.medcle.2018.03.028 29705155

[jocn17226-bib-0065] Vernooij, J. W. , Kaasjager, H. A. , van der Graaf, Y. , Wierdsma, J. , Grandjean, H. M. , Hovens, M. M. , de Wit, G. A. , & Visseren, F. L. (2012). Internet based vascular risk factor management for patients with clinically manifest vascular disease: Randomised controlled trial. BMJ, 344, e3750. 10.1136/bmj.e3750 22692651 PMC3374126

[jocn17226-bib-0066] Wakefield, B. J. , Holman, J. E. , Ray, A. , Scherubel, M. , Burns, T. L. , Kienzle, M. G. , & Rosenthal, G. E. (2009). Outcomes of a home telehealth intervention for patients with heart failure. Journal of Telemedicine and Telecare, 15(1), 46–50. 10.1258/jtt.2008.080701 19139220

[jocn17226-bib-0067] Wakefield, B. J. , Koopman, R. J. , Keplinger, L. E. , Bomar, M. , Bernt, B. , Johanning, J. L. , Kruse, R. L. , Davis, J. W. , Wakefield, D. S. , & Mehr, D. R. (2014). Effect of home telemonitoring on glycemic and blood pressure control in primary care clinic patients with diabetes. Telemedicine and e‐Health, 20(3), 199–205. 10.1089/tmj.2013.0151 24404819 PMC3934666

[jocn17226-bib-0068] Wakefield, B. J. , Ray, A. , Scherubel, M. , Adams, M. R. , Hillis, S. L. , & Rosenthal, G. E. (2011). Effectiveness of home telehealth in comorbid diabetes and hypertension: A randomized, controlled trial . 10.1089/tmj.2010.0176 21476945

[jocn17226-bib-0069] Walker, R. C. , Tong, A. , Howard, K. , & Palmer, S. C. (2019). Patient expectations and experiences of remote monitoring for chronic diseases: Systematic review and thematic synthesis of qualitative studies. International Journal of Medical Informatics, 124, 78–85. 10.1016/j.ijmedinf.2019.01.013 30784430

[jocn17226-bib-0070] Wang, J. , Tong, Y. , Jiang, Y. , Zhu, H. , Gao, H. , Wei, R. , Que, X. , & Gao, L. (2018). The effectiveness of extended care based on internet and home care platform for orthopaedics after hip replacement surgery in China. Journal of Clinical Nursing (John Wiley & Sons, Inc.), 27(21–22), 4077–4088. 10.1111/jocn.14545 29851157

[jocn17226-bib-0071] Wheelock, A. E. , Bock, M. A. , Martin, E. L. , Hwang, J. , Ernest, M. L. , Rugo, H. S. , Esserman, L. J. , & Melisko, M. E. (2015). SIS.NET: A randomized controlled trial evaluating a web‐based system for symptom management after treatment of breast cancer. Cancer, 121(6), 893–899. 10.1002/cncr.29088 25469673

[jocn17226-bib-0072] WHO . (2018). Health and Sustainability Development, World Health Organisation, (http://www.who.int/sustainable‐development/health‐sector/strategies/telehealth/en/) Accessed at: 22.04.2018.

[jocn17226-bib-0073] Willems, D. C. M. , Joore, M. A. , Hendriks, J. J. E. , Nieman, F. H. M. , Severens, J. L. , & Wouters, E. F. M. (2008). The effectiveness of nurse‐led telemonitoring of asthma: Results of a randomized controlled trial. Journal of Evaluation in Clinical Practice, 14(4), 600–609. 19126178 10.1111/j.1365-2753.2007.00936.x

[jocn17226-bib-0074] Wong, A. K. C. , Wong, F. K. Y. , Bayuo, J. , Chow, K. K. S. , Wong, S. M. , & Lee, A. Y. L. (2022). A randomized controlled trial of an mHealth application with nursing interaction to promote quality of life among community‐dwelling older adults. Frontiers in Psychiatry, 13, 978416. 10.3389/fpsyt.2022.978416 36329920 PMC9623156

[jocn17226-bib-0075] Yan, W. , Liu, L. , Huang, W. Z. , Wang, Z. J. , Yu, S. B. , Mai, G. H. , Meng, M. M. , & Cui, S. Y. (2022). Study on the application of the internet + nursing service in family rehabilitation of common bone and joint diseases in the elderly. European Review for Medical and Pharmacological Sciences, 26(18), 6444–6450. 10.26355/eurrev_202209_29743 36196694

[jocn17226-bib-0076] Zhang, Q. , Li, F. , Zhang, H. , Yu, X. , & Cong, Y. (2018). Effects of nurse‐led home‐based exercise & cognitive behavioral therapy on reducing cancer‐related fatigue in patients with ovarian cancer during and after chemotherapy: A randomized controlled trial. International Journal of Nursing Studies, 78, 52–60. 10.1016/j.ijnurstu.2017.08.010 28939343

