# Supplementary material for: Nurse‐led remote digital support for adults with chronic conditions: A systematic synthesis without meta‐analysis
Source: J Clin Nurs. 2024 Jun 18;34(3):715–36. doi: 10.1111/jocn.17226 (PMC11808464; doi:10.1111/jocn.17226)
Supplement: Supplementary file 2 — Appendix S2. [file JOCN-34-715-s001.docx]

**Supplementary File One**

MEDLINE Search Strategy

| **#** | **Searches** |
| --- | --- |
| 1 | exp nurses/ and (based or led or lead* or manage* or coordinat* or co-ordinat*).tw,kf. |
| 2 | (nurse? adj2 based).tw,kf. |
| 3 | (nurse? adj3 (led or lead* or manage* or coordinat* or co-ordinat*)).tw,kf. |
| 4 | or/1-3 |
| 5 | Telemedicine/ |
| 6 | Telephone/ |
| 7 | Cell phone/ |
| 8 | Smartphone/ |
| 9 | Mobile Applications/ |
| 10 | Electronic Mail/ |
| 11 | Text Messaging/ |
| 12 | Internet/ |
| 13 | Internet-Based Intervention/ |
| 14 | (electronic health or ehealth or "e-health" or mobile health or mhealth or "m-health" or telehealth or tele-health or telemedicine or tele-medicine).tw,kf. |
| 15 | (intervention? adj5 (email* or "e-mail*" or internet or mobile? or online or personal digital assistant? or PDA? or phone? or smartphone? or tablet? or web-based or wireless device?)).tw,kf. |
| 16 | (device? adj2 (patient monitor* or monitor* patient? or physiolog* monitor* or monitor* physiolog*)).tw,kf. |
| 17 | or/5-16 |
| 18 | 4 and 17 |
| 19 | ("20200319" or "20200320" or "20200321" or "20200322" or "20200323" or "20200324" or "20200325" or "20200326" or "20200327" or "20200328" or "20200329" or "20200330" or "20200331" or 202004* or 202005* or 202006* or 202007* or 202008* or 202009* or 202010* or 202011* or 202012* or 2021*).dt,ez,da. |
| 20 | 18 and 19 |
